# Quantitative Optical Coherence Tomography Angiography Biomarkers in a Treat-and-Extend Dosing Regimen in Neovascular Age-Related Macular Degeneration

**DOI:** 10.1167/tvst.9.3.18

**Published:** 2020-02-14

**Authors:** Diogo Cabral, Florence Coscas, Telmo Pereira, Catherine Français, Carlos Geraldes, Rita Laiginhas, Catarina Rodrigues, Alexis Khorrami Kashi, Vanda Nogueira, Manuel Falcão, Ana Luísa Papoila, Marco Lupidi, Gabriel Coscas, Salomon Yves Cohen, Eric Souied

**Affiliations:** 1 Centre Ophtalmologique de l'Odéon, Paris, France; 2 CEDOC, NOVA Medical School I Faculdade de Ciências Médicas, Universidade NOVA de Lisboa, Portugal; 3 Instituto de Oftalmologia Dr. Gama Pinto, Lisboa, Portugal; 4 Department of Ophthalmology, Centre Hospitalier Intercommunal de Creteil, University Paris Est Creteil XII, Creteil, France; 5 Centro Hospitalar de Entre o Douro e Vouga; Faculty of Medicine of Porto University. Porto, Portugal; 6 Centro Hospitalar de São João; Department of Surgery and Physiology, Faculty of Medicine of Porto University, Porto, Portugal; 7 Department of Surgical and Biomedical Sciences, Section of Ophthalmology, University of Perugia, S. Maria della Misericordia Hospital, Perugia, Italy

**Keywords:** age-related macular degeneration, treat-and-extend protocol, choroidal neovascularization, optical coherence tomography angiography, anti-vascular endothelial growth factor

## Abstract

**Purpose:**

To evaluate the association between quantitative optical coherence tomography angiography (OCT-A) parameters and clinical outcomes in treatment-naïve neovascular age-related macular degeneration (nAMD) patients treated with a treat-and-extend dosing regimen on a 12-month follow-up interval.

**Methods:**

Observational, prospective study of consecutive patients. The treatment protocol was based on a loading dose of three anti-vascular endothelial growth factor (VEGF) intravitreal injections (IVI) followed by a treat-and-extend regimen. Eyes were evaluated by swept-source OCT-A at baseline, 1 month after the loading dose and at 12 months. A quantitative analysis was issued for fractal dimension (FD), lacunarity index (LAC), blood flow surface area (SA), and vessel density (VD). An association of these parameters with the anatomic response and functional responses, and IVI number at 12 months of follow-up was assessed. A level of significance α = 0.05 was considered.

**Results:**

Sixty-four patients were included, 52 of whom (81%) completed the 12-month study protocol. The median number of injections at 12 months was 7 (P_25_-P_75_: 6-12). FD and SA were reduced 1 month after the loading dose of anti-VEGF (*P* < 0.001). The generalized linear models using baseline FD and baseline SA achieved the best performance in discriminating a lower treatment burden (area under the curve [AUC] = 0.78; 95% confidence interval [CI]: 0.64–0.91 and AUC = 0.76; 95% CI: 0.63–0.90, respectively).

**Conclusions:**

Baseline OCT-A may provide useful biomarkers for the treatment burden in nAMD.

**Translational Relevance:**

The application of fractal dimension and automatic blood flow area algorithms to OCT-A data can distinguish patients with distinct treatment burdens in the first year of nAMD.

## Introduction

Late stage age-related macular degeneration (AMD) has been considered as the leading cause of blindness in industrialized countries.^1^ In most cases, neovascular AMD (nAMD) is characterized by the growth of abnormal blood vessels from the choroid through Bruch's membrane. The natural course of the disease is characterized by exudation, disruption of the outer retinal architecture, and fibrosis leading to an irreversible vision loss.^2^ Clinical trials have proven that regular intravitreal injections (IVI) of vascular endothelial growth factor (VEGF) inhibitors reduce the exudation and slow-down the progression of the disease.^3^ The burden of regular IVI dictated the need to adapt treatment protocols following the rapid initial functional and structural response to anti-VEGF IVI.^4^ In daily clinical practice, the effect of anti-VEGF therapy is efficiently monitored with non-invasive structural optical coherence tomography (OCT). This follow-up relies on indirect signs of neovascular activity, such as intraretinal fluid, subretinal fluid, or pigment epithelial detachments.^3^

OCT-angiography (OCT-A) is the most recent advance of OCT technology and provides high-resolution images of retinal and choroidal vascular perfusion.^5^ It can detect choroidal neovascularization (CNV) with the same sensitivity as dye angiographies and enables the evaluation of morphological features of neovascular networks.^6^ Recently, some authors have demonstrated consistent qualitative features and immature lesions were associated with a greater rate of small branching vessels and peripheral arcades and hypermature lesions with a dead-tree appearance.^6^^–^^8^

To eliminate the subjectivity of the qualitative evaluation, there has been a growing interest on quantitative features of neovascular networks, and the evaluation of novel imaging biomarkers for nAMD disease activity has become a topic of increasing research interest. It has been previously demonstrated that the CNV surface area (SA)^9^ and vessel density (VD)^10^ could be reliably measured during the follow up and that the branching complexity and organization of the neovascular network could be characterized using fractal-based metrics, such as fractal dimension (FD)^11^^,^^12^ and lacunarity (LAC).^13^

Changes in blood flow area following the loading dose and during “pro re nata” treatment with anti-VEGF have been described.^7^^,^^9^ These studies showed that the blood flow area increased during the follow-up, however they were not able to identify an association between area modifications and exudation. The characterization of quantitative OCT-A biomarkers during a treat and extend protocol remains to be established. The aim of this study was to evaluate quantitative OCT-A parameters of CNVs under a treat and extend (TAE) dosing regimen over a twelve-month follow-up period and to study their association with clinical outcomes.

## Material and Methods

### Study Design and Setting

Prospective, observational real-life study of consecutive patients observed during routine clinical practice at the *Centre Ophtalmologique de l'Odeon* (Paris, France) between June 2017 and June 2019. This study had institutional review board approval from Paris Est University and was conducted in accordance with the tenets of the Declaration of Helsinki (1964) and the French legislation. All the enrolled patients gave their written informed consent at the time of the study recruitment.

### Participants

Patients with a novel diagnosis of exudative AMD (either type 1 or type 2 CNV) were enrolled. The classification of CNV subtype was based on multimodal imaging (OCT, fluorescein angiography, and indocyanine green angiography) and was independently reviewed by two experienced examiners (F.C., C.F.). Only one eye per patient could enter the study (in cases of bilateral disease, the first eye diagnosed with nAMD was enrolled). Exclusion criteria were: spherical equivalent higher than +3 or lower than -6.0 diopters; diagnosis of glaucoma or ocular hypertension; other retinal confounding diseases complicated with secondary neovascularization; poor quality images in OCT-A (signal strength index <80); evident motion artifacts; neovascular network that exceeded the 4.5 × 4.5 mm scanning area; multiple CNV lesions; pigment epithelium detachment (PED) height > 250 µm; and central geographic atrophy.

### Treatment and Observation Protocol

The treatment protocol consisted in a loading dose of 3 monthly IVI of anti-VEGF (aflibercept) after the nAMD diagnosis followed by a TAE treatment protocol. Patient follow-up visits and treatments were extended by intervals of two weeks if no signs of CNV activity were present on structural-OCT scans. However, if any signs of neovascular activity were detected, treatment intervals were subsequently shortened by a 2-week interval. Anti-VEGF treatment was administered at every visit, regardless of CNV activity. Neovascular activity was considered in the presence of one of the following criteria: visual acuity loss of at least five letters with structural-OCT evidence of intraretinal and/or subretinal fluid within the macula; macular hemorrhage; evidence of persistent or increased fluid accumulation on structural OCT after the previous injection.^4^

### Data Collection

All patients had comprehensive medical observations scheduled according to the treatment protocol criteria. Data were collected at the time of diagnosis (baseline), 1 month after the loading dose (3-month visit) and 12 months after the first IVI (12-month visit). In each visit, the following procedures were performed: best corrected visual acuity (BCVA) using the Early Treatment of Diabetic Retinopathy Study (ETDRS) visual chart, color fundus photography, spectral domain structural OCT-B scans (Spectralis OCT, Heidelberg Engineering Inc., Heidelberg, Germany) and swept-source OCT-A (DRI OCT Triton, Topcon, Tokyo, Japan). At baseline, all patients performed two consecutive measurements of FD, LAC, SA, and VD for repeatability analysis. The first acquisition was used for further analysis. Pertinent clinical characteristics were recorded for statistical analyses.

### Analysis of Structural and Clinical Outcomes

The anatomic response after the loading dose was classified as either good response or bad response. Because a standardized definition of good responders versus bad responders has not yet been validated, we defined the anatomic response based on previous reports.^4^^,^^14^^,^^15^ A good responder was defined as the complete resolution of the intraretinal or subretinal fluid or more than 100 µm decrease of central macular thickness 1 month following the last IVI of the loading dose. A bad responder was defined as having an increase of central macular thickness or a decrease of less than 100 µm in the same time point.

A good functional response was defined as a gain in the BCVA of at least five ETDRS letters between baseline and the 12-month visit.

A subanalysis regarding the treatment burden was performed according to the number of IVI received during the 12-month follow-up. Cutoff values were established on the base of the average number of treatments that are expected during 12 months of TAE.^16^ Accordingly, regular treatment was considered for patients with 8 or more IVI and extended treatment was considered for patients with 7 or less injections in the first 12 months.

A diagram of the observation protocol including the study outcomes is available as a supplementary Figure S1.

### Image Acquisition and Analysis

**Figure 1.**
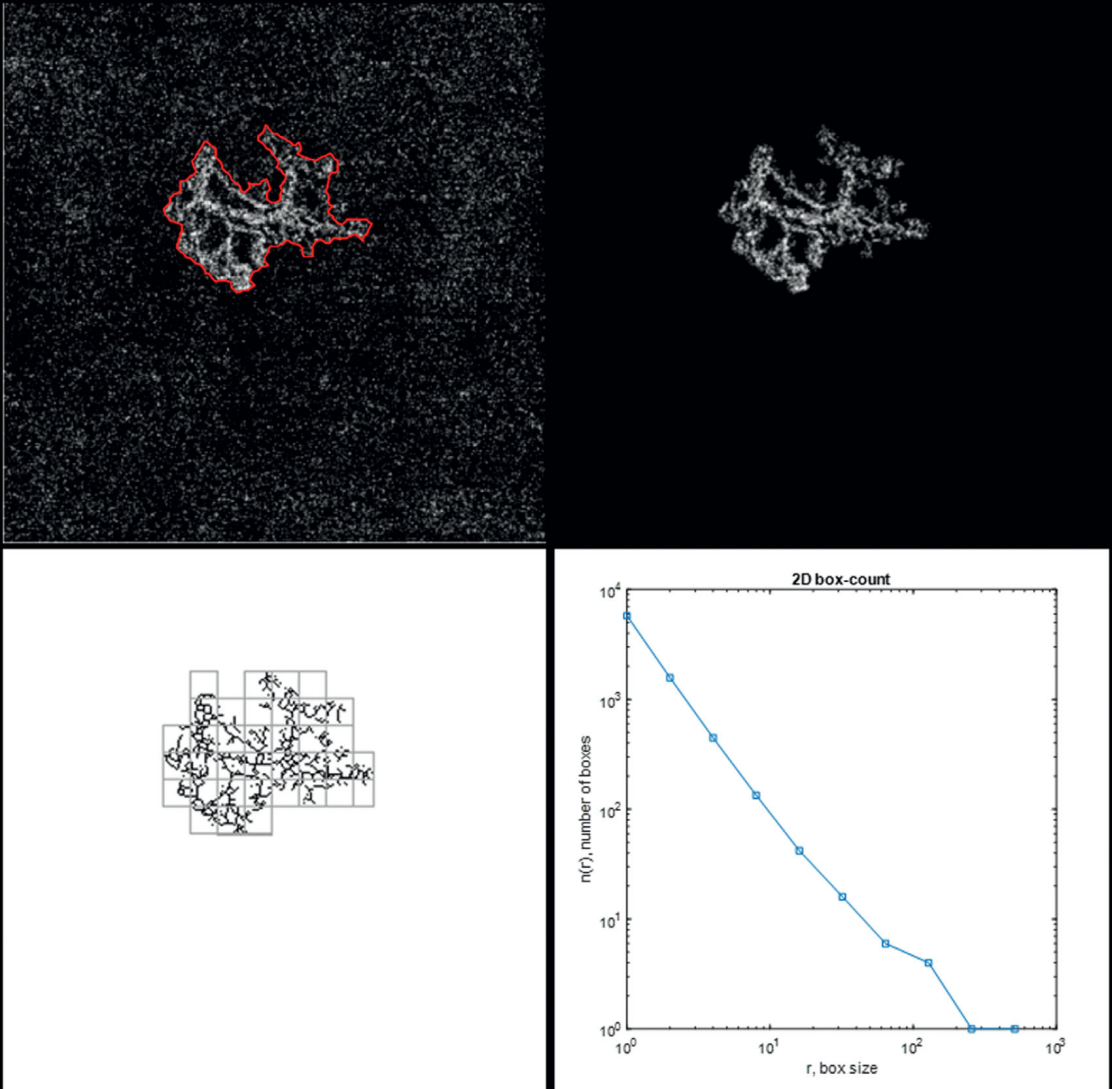
Composite exposing the image analysis protocol. (*Upper left*) Optical coherence tomography angiography (OCTA) 4.5 × 4.5 mm outer retina and choriocapillaris slab showing the automatic delimitation of the choroidal neovascularization (CNV). (*Upper right*) Blood flow is isolated from the background for further analysis. (*Lower left*) Skeletonization of the blood flow aspect and division of the image by squares of equal sizes. (*Lower right*) The process is repeated by a power series of box sizes (base = 2 ; exponent = 2); a graphic with the output of the box-counting method is displayed: N (the number of boxes needed to cover the set) is depicted as a function of R (the size of the boxes); as the set is a fractal, we can observe a power-law relationship [N = N0 _ R^(-DF)], with DF being the fractal dimension (Kolmogorov capacity).

Enhanced depth imaging spectral domain OCT with a macular volume scan of 49 B-scans and a 30 degrees line scan centered on the fovea were performed using OCT 2 and the Heyex v6.9a software (Spectralis OCT). Optical coherence tomography angiography images of 4.5 × 4.5mm centered on the fovea were obtained with the swept-source DRI OCT Triton (Topcon, Tokyo, Japan). IMAGEnet 6 version 1.25 was used to evaluate the outer retinal layers, between the outer plexiform layer and Bruch's membrane, for detecting potential blood flow abnormalities suggestive of CNV.^17^ Projection artifacts were subtracted automatically using the inbuilt algorithm. Images were exported as tagged image file format (TIFF). OCT-A image analysis was done using a previously validated custom graphical user interface built in MATLAB (v. r2018a) coding language that enables to detect blood flow automatically.^12^ SA and VD were calculated from binary images. The box counting method starting at multiple origins was applied to the image of the binary skeleton to estimate FD^18^ and LAC^19^ of the vascular network, which are global indices of morphological complexity and structural nonuniformity, respectively.^20^ Box sizes followed the power of two series until a box of half image pixel size was reached. The results were automatically exported into a comma separated file for further analysis. A composite about the image analysis protocol is presented in Figure 1.

### Statistical Analysis

An exploratory study of demographic, clinical, and OCT-A measurements (FD, LAC, SA, and VD) was performed. Continuous variables were presented as mean and standard deviation (SD) or median and interquartile range (25th-75th percentile) or range (R: minimum:maximum), as appropriate.

To assess reliability of measurements, the intraclass correlation coefficient (ICC) between the two consecutive baseline acquisitions was estimated based on a mean-rating (k = 2), absolute-agreement, 2-way mixed-effects model.

Intragroup serial comparisons were carried out using the exact Wilcoxon test or sign test, as appropriate. Intergroup comparisons were preformed using Mann-Whitney test.

We analyzed the association between each of the OCTA measurements and the binary dependent variables: treatment response after the loading dose (good response vs. bad response to anti-VEGF), functional response (gain in the BCVA of at least five ETDRS letters vs the remaining patients) and treatment burden (less than eight IVIs vs eight or more IVIs). Generalized linear models (GLM)^21^ for binary response were used for this purpose. Receiver operating characteristic curves were used to evaluate the discriminative ability of each model. Generally, an area under the curve (AUC) value above 0.80 indicated a very good discriminative ability, a value between 0.70 and 0.80 a good ability, and a value between 0.50 and 0.70 a weak discriminative ability. A *P* value ≤0.05 was considered as statistically significant. Statistical analyses were performed using both IBM SPSS Statistics for windows, version 25.0 (Armonk, NY: IBM Corp.) and R (version 3.6.0; R Foundation for Statistical Computing).

## Results

During screening, 79 patients were considered eligible. From this cohort were excluded: 9 patients because of PED detachment height superior to 250 µm; 4 patients from imaging artifacts; 1 patient because of concurring central geographic atrophy; and 1 patient because of high spherical equivalent. Therefore, a total of 64 patients were considered at baseline, all of them had CNV blood flow detectable using Swept-Source (SS)-OCTA. From this cohort, 52 (81%) completed the 12-month study protocol. Regarding patients that did not complete the 12-month follow up, information from the first medical visit and the visit after the loading of anti-VEGF were used (clinical and demographical characteristics are detailed in Table 1).

**Table 1.** Demographic and Clinical Characteristics at Baseline (n = 64)

Sex	
Male, n (%)	33 (52)
Female, n (%)	31 (48)
Age, mean (SD), years	78.5 (8.1)
Eye	
Right, n (%)	29 (45)
Left, n (%)	35 (55)
Type of CNV	
1, n (%)	38 (59)
2, n (%)	26 (41)
Central macular thickness, mean (SD), µm	390 (116)
BCVA, median (range), (ETDRS letters)	60 (20-85)

SD, standard deviation.

**Figure 2.**
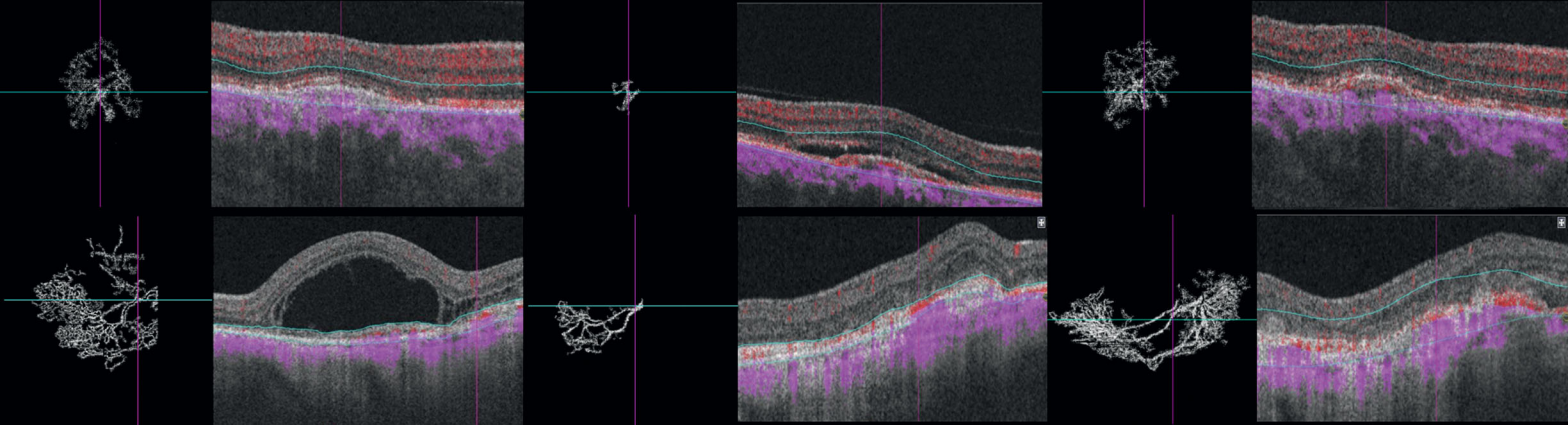
Treatment-naïve choroidal neovascularization (CNV) in AMD before and after intravitreal anti-VEGF injections. (*Upper row*) The right eye of a 77-year-old male patient with type 2 CNV and a baseline visual acuity of 20/100. (*Upper left*) Optical coherence tomography angiography (OCTA) image of the neovascular complex after automatic delineation. We observe a densely branched network (SA = 1.68 mm^2^, FD = 1.51) with tiny capillaries emanating from the center of the lesion and subretinal fluid in the structural B-scan OCT. (*Upper middle*) Follow-up OCTA 4 weeks after the loading dose; the visual acuity was 20/60. We observe the pruning effect of anti-VEGF, with disappearance of the tiny vessels, a voluminous central core vessel (SA = 0.13 mm^2^, FD = 1.31) and the absence of intraretinal fluids in structural OCT. (*Upper right*) Follow-up OCTA 12 months after the first treatment and after a total of 7 intravitreal injections given following a treat-and-extend protocol. The visual acuity improved to 20/40. In the OCTA, we observe a regrowth of a branching network from the central core (SA = 1.71 mm^2^, FD = 1.54). In the structural OCT, we noticed a nasal growth of the CNV and a small lamella of subretinal fluid. (*Lower row*) The left eye of a 79-year-old male patient with type 2 CNV and a baseline visual acuity of 20/125. (*Lower left*) OCTA of the neovascular complex after automatic delineation. We observe a densely branched network with loops and tiny capillaries emanating from the center of the lesion (SA = 4.65 mm^2^, FD = 1.53) and massive subfoveal fluid in the structural B-scan OCT. (*Lower middle*) Follow-up OCTA en face projection 4 weeks after the loading dose; the visual acuity was 20/63. We observe the pruning effect of anti-VEGF, with a disappearance of the tiny vessels, the presence of a voluminous central core vessel, few anastomosed vessels (SA = 2.36 mm^2^, FD = 1.44), and the absence of intraretinal fluids in structural OCT. (*Lower right*) Follow-up OCTA 12 months after the first treatment and after a total of 6 intravitreal injections given following a treat-and-extend protocol. The visual acuity remained 20/63. In the OCT-A, we observe the regrowth of tiny vessels from the central core with an expansion of the CNV surface area (SA = 3.94 mm^2^, FD = 1.54). In the structural OCT, we notice the development of subretinal fibrosis with erosion of the outer retinal layer architecture.

Regarding OCT-A parameters, FD and SA decreased between baseline and the 3-month visit (*P* < 0.001 and *P* = 0.006, respectively). Afterwards, we observed a regrowth of the lesion and at the 12-month visit the median values of OCTA parameters were not statistically different from baseline values (Table 2). Figure 2 represents examples of typical active neovascularization lesions in which we can observe the pruning effect of anti-VEGF after the loading dose of anti-VEGF, with disappearance of the tiny vessels along with a shrinkage of blood flow surface area, and the regrowth of the CNV observed at the 12-month visit. A complete characterization of clinical and OCT-A parameters during the follow-up is available in Supplementary Table S1.

**Table 2.** OCT-A Parameters at Different Follow-Up Evaluations

	Baseline	3 M^*^	*P* ^†^	12M	*P* ^‡^
FD	1.49 (1.40-1.55)	1.44 (1.25-1.53)	<0.001	1.47 (1.37-1.54)	0.198
LAC	0.29 (0.26-0.34)	0.29 (0.26-0.33)	0.669	0.32 (0.27-0.35)	0.126
SA	0.62 (0.28-1.52)	0.47 (0.14-1.43)	0.006	0.92 (0.35-2.08)	0.757
VD	0.55 (0.49-0.67)	0.50 (0.42-0.60)	0.062	0.53 (0.44-0.61)	0.092

Results are presented as median (interquartile range).

* One month after the last of 3 monthly injections.

† *P* value for the comparison of baseline and 3-month values (n = 64).

‡ *P* value for the comparison of baseline and 12-month values (n = 52). M = months.

Medians comparison and the discriminative performance analyses of univariable models regarding each OCT-A parameter (FD, LAC, SA, and VD) for each outcome is summarized in Supplementary Tables S2-S4 and Supplementary Tables S5-S7, respectively.

A good anatomic response after the loading dose was verified in 63% (40/64) of patients. OCT-A parameters medians comparison did not disclose any significant difference and none of the GLM models attained a relevant discriminative ability.

**Figure 3.**
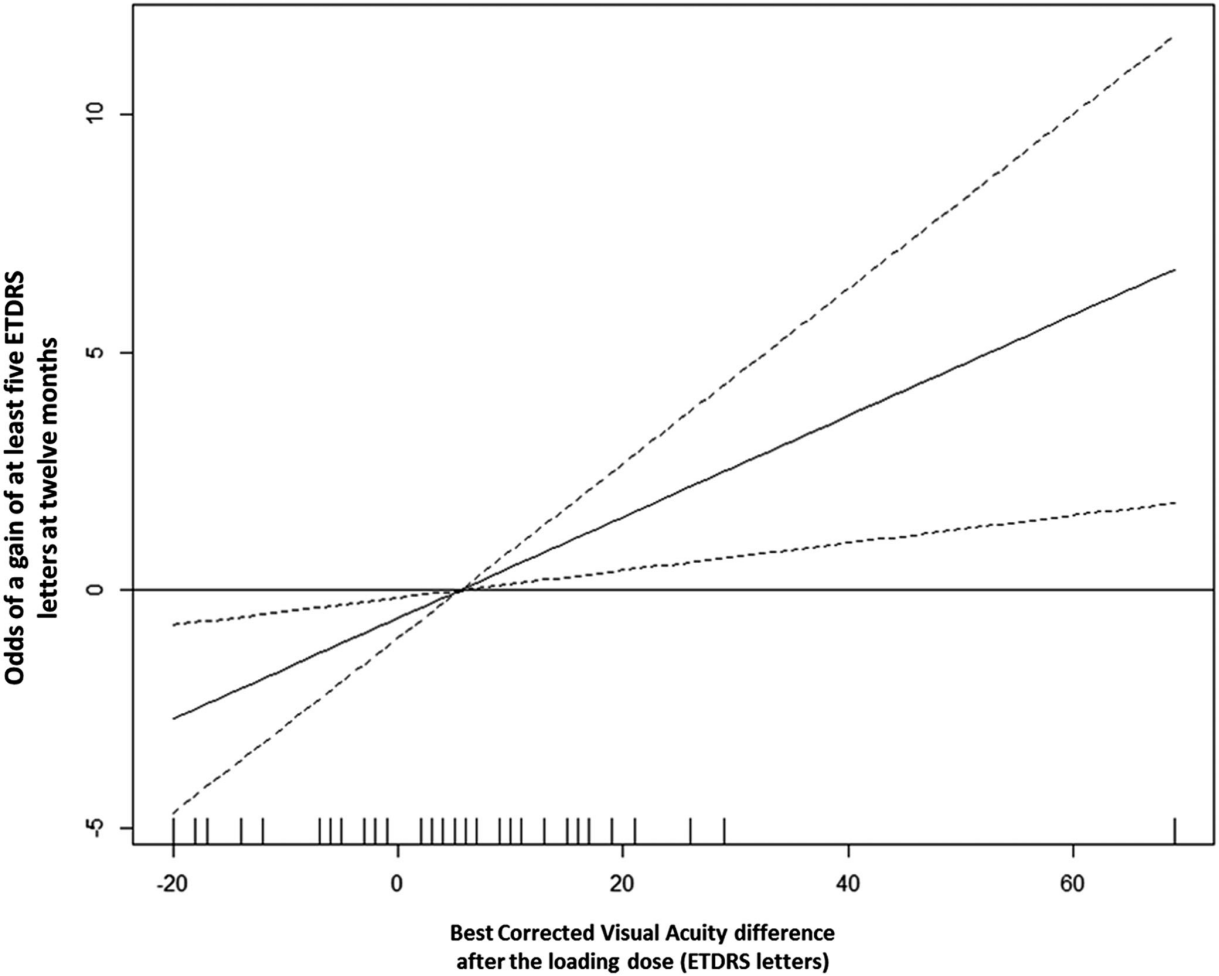
Partial function showing the functional form of the association between the BCVA difference after loading dose (x-axis) and the odds of a gain of at least 5 ETDRS letters at 12 months (y-axis). The solid curve represents the estimate of this functional form; the dashed curves represent the lower and upper limits of the 95% confidence intervals estimated for each BCVA difference after the loading dose. Positive values on the y-axis mean higher odds of a good functional outcome (gain of at least 5 ETDRS letters) at 12 months.

A good functional response was present in 63% (33/52) of patients. OCTA parameters medians comparison did not disclose any significant difference and none of the GLM models attained a relevant discriminative ability. The median BCVA difference after loading dose to baseline was 7 (4-15) in patients with a good functional response and in bad responders was -1 (-6 to 7) ETDRS letters (*P* = 0.001). A GLM model using this parameter achieved a good performance in identifying patients with a good functional response at 12 months (AUC = 0.78; 95% confidence interval [CI], 0.64-0.92). The odds ratio estimate was 1.11 (95% CI, 1.04-1.21). The association between BCVA difference after loading dose and a good functional response at 12 months is depicted in Figure 3.

**Figure 4.**
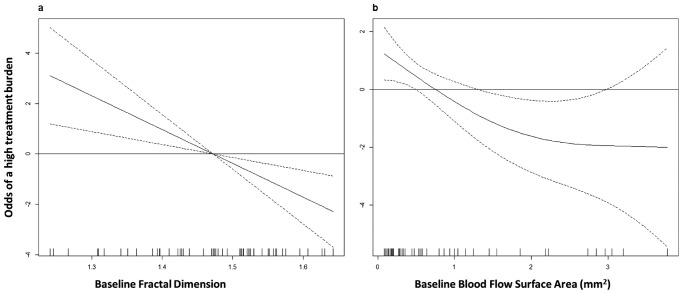
Partial functions showing the functional form of the association between the baseline FD (x-axis, A) or baseline blood flow surface area (x axis, B) and the treatment burden (y-axis). The solid curve represents the estimate of this functional form, the dashed curves represent the lower and upper limits of the 95% confidence intervals estimated for each FD unit (A) or baseline blood flow surface area (B). Positive values in the y-axis mean higher odds of a high treatment burden (defined as at least 8 IVI in the first year of treatments).

**Figure 5.**
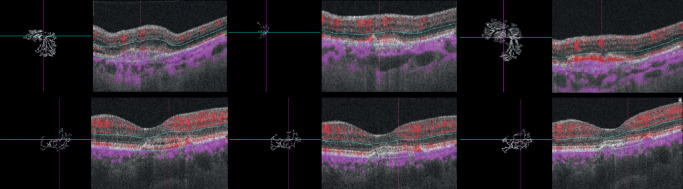
Treatment-naïve CNV in AMD with a low treatment burden of intravitreal anti-VEGF injections. (*Upper* *row*) The left eye of an 81-year-old male patient with type 1 CNV and a baseline visual acuity of 20/25. (*Upper left*) OCT-A of the neovascular complex after automatic delineation. We observe a medium sized branched and organized network (SA = 1.14 mm^2^, FD = 1.51) and pigment epithelium detachments (PED) with subretinal fluid in the structural B-scan OCT. (*Upper* *middle*) Follow-up OCT-A en face projection 4 weeks after the loading dose, the visual acuity remained stable (20/25). We observe a reduction of blood flow area and of the branching pattern, only central vessels remain (SA = 0.77 mm^2^, FD = 1.32) and there is an absence of fluids in structural OCT. (*Upper* *right*) Follow-up OCT-A 12 months after the first treatment and after a total of 7 intravitreal injections given following a treat-and-extend protocol. The visual acuity remained stable (20/25). In the OCT-A blood flow, we observed a growth of the CNV and an organized and branched network around two main trunks (SA = 2.33 mm^2^, FD = 1.57). In the structural OCT, we noticed a reduction of the height with an increase of the extension of the PED and an absence of subretinal fluid. (*Lower* *row*) The left eye of a 66-year-old female patient with type 1 CNV and a baseline visual acuity of 20/40. (*Upper left*) OCT-A image of the neovascular complex after automatic delineation. We observed an organized branched network of anastomosed linear capillaries (SA = 0.63 mm^2^, FD = 1.48) and subretinal fluid in the structural B-scan OCT. (*Upper middle*) Follow-up OCT-A 4 weeks after the loading dose; visual acuity improved to 20/20. Blood flow aspect remains unchanged (SA = 0.67 mm^2^, FD = 1.48) and we observed the persistence of a small lamella of subretinal fluid in structural OCT. (*Upper right*) Follow-up OCTA 12 months after the first treatment and after a total of 6 intravitreal injections given following a treat-and-extend protocol. The visual acuity remained 20/20. In the OCT-A, we observed a small increase in SA and branching complexity of the CNV blood flow (SA = 0.82 mm^2^, FD = 1.49) and the persistence of a residual lamella of subretinal fluid in structural OCT.

**Figure 6.**
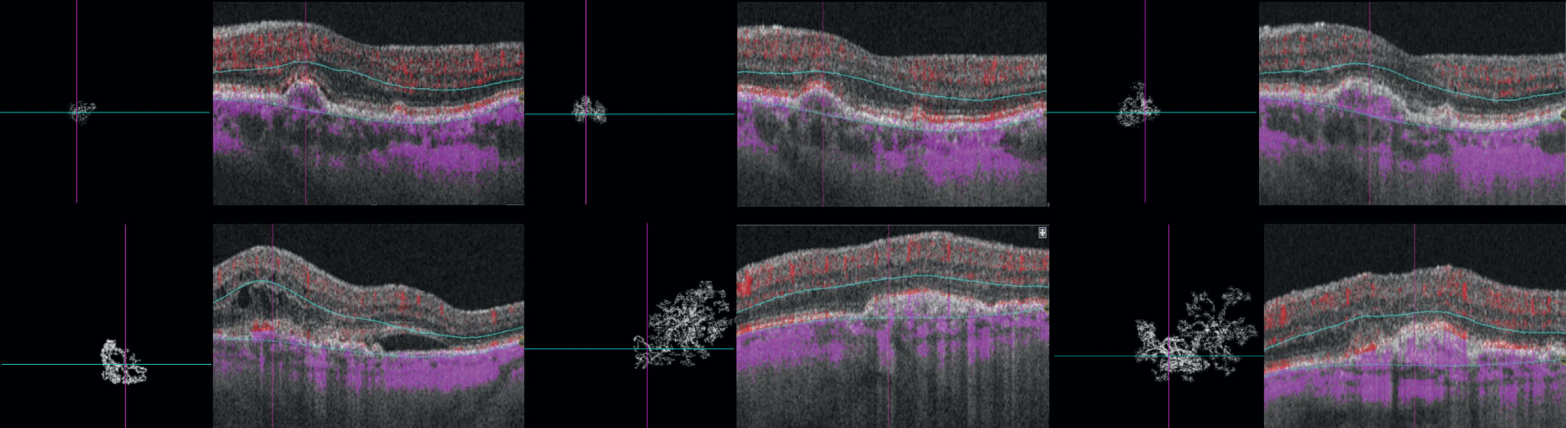
Treatment naïve CNV in AMD with a high treatment burden of intravitreal anti-VEGF injections. (*Upper* *row*) The left eye of an 82-year-old male patient with type 1 CNV and a baseline visual acuity of 20/25. (*Upper left*) OCT-A of the neovascular complex after automatic delineation. We observed a small, densely branched and disorganized network (SA = 0.15 mm^2^, FD = 1.24) and small pigment epithelium detachments (PED) with subretinal fluid in the structural B-scan OCT. (*Upper* *middle*) Follow-up OCTA en face projection 4 weeks after the loading dose; visual acuity remained 20/25. We observed a circumferential growth of the lesion with reorganization of the blood flow around a central core vessel (SA = 0.26 mm^2^, FD = 1.51) and the absence of fluids in structural OCT. (*Upper* *right*) Follow-up OCT-A 12 months after the first treatment and after a total of 11 intravitreal injections given following a treat-and-extend protocol. The visual acuity has dropped to 20/40. In the OCT-A, we observed the definition of a central core vessel with tiny capillaries emanating toward the nasal half of the lesion (SA = 0.44 mm^2^, FD = 1.48). In the structural OCT, we noticed an increase of the extension of a fibrovascular PED and subretinal fluid. (*Lower* *row*) The left eye of an 84-year-old male patient with type 2 CNV and a baseline visual acuity of 20/63. (*Lower left*) OCT-A of the neovascular complex after automatic delineation. We observed a small and densely branched network with small vessels emanating from a central core lesion (SA = 0.48 mm^2^, FD = 1.35) and intraretinal cysts in the structural B-scan OCT. (*Lower* *middle*) Follow-up OCT-A en face projection 4 weeks after the loading dose with visual acuity of 20/32. We observed a circumferential growth of the lesion, a densely anastomotic network (SA = 2.32 mm^2^, FD = 1.60) and the absence of fluids in structural OCT. (*Lower right*) Follow-up OCT-A 12 months after the first treatment and after a total of 10 intravitreal injections given following a treat-and-extend protocol. The visual acuity has dropped to 20/63. In the OCT-A, we observe the organization of the blood flow aspect with anastomotic loops in the nasal half and linear capillaries in the temporal half of the lesion (SA = 2.66 mm^2^, FD = 1.56). In the structural OCT, we noticed an increase of the height of the fibrovascular PED, hyperreflective intraretinal dots above the PED, and the absence of retinal fluids.

Regarding treatment burden, the median number of injections at 12 months was 7 (range: 6-12); 44% (n = 23) of the patients were classified as regular treatment. All patients had at least some response to the drug administered, so we had no cases of switching. The median baseline FD in regular treatment was 1.40 (1.32-1.49) and in extension treatment was 1.52 (1.47-1.56) (*P* = 0.001). The median baseline SA in regular treatment patients was 0.33 (0.17-0.58) and in the extension treatment was 1.14 (0.47-2.79) (*P* = 0.001). GLM models using baseline FD (AUC = 0.78; 95% CI, 0.64-0.91) and baseline SA (AUC = 0.76; 95% CI, 0.63-0.90) achieved the best performance. The odds ratio estimate of baseline FD was 0.99 (95% CI, 0.98-0.99) and of baseline SA was 0.29 (95% CI, 0.09-0.68). The association between baseline FD and baseline SA with treatment burden may be observed in Figure 4. Figures 5 and 6 represent typical examples that reflect the association between baseline FD and baseline SA with treatment burden. We can observe that CNVs with a higher FD or a higher SA at baseline had a lower treatment burden (seven of less IVI) than lesions with a lower FD, that required at least eight IVI during the first year of treatments.

### Repeatability Evaluation

The means of the different parameters measured together with the ICC are listed in Supplementary Table S8. ICC for all the parameters analyzed were above 0.90.

## Discussion

In this study, with an attempt of a novel approach, used OCTA to monitor quantitative flow changes in treatment-naïve CNV during 1 year of anti-VEGF treatment under a TAE regimen. The branching complexity and blood flow area decrease immediately after the loading dose and return to the original size at 12 months. We highlighted several quantitative features of CNV blood flow before the anti-VEGF treatment that might be discriminative of the number of IVIs in the first year of a TAE regimen.

Previous qualitative assessments of OCT-A images in CNV networks showed that most of the lesions demonstrated shrinkage of fine peripheral vessels and arteriogenesis of the remaining vessels after anti-VEGF treatment.^22^ Coscas et al. found an area reduction in mixed type 1 and 2 neovascularization after 4 weeks of VEGF trap treatment.^23^ More recently, other studies have studied FD and LAC as potential quantitative biomarkers to characterize CNV branching complexity and heterogeneity, respectively.^11^^–^^13^

FD quantifies the variations in space filling using the box-counting method and provides a way of characterizing branching in terms of complexity. In two-dimensional images, FD attains a value between zero and two with higher values indicating increased pattern complexity. Previous works demonstrated that FD varies according to the number of secondary divisions of the CNV: the higher the number of discernible secondary divisions, the higher the FD value.^11^^,^^12^ Al-Sheik et al. evaluated eyes with non-naïve active CNV and qualitative features of angiogenesis following one anti-VEGF IVI. They found attenuation and pruning of secondary ramifications in en-face OCTA, with subsequent reduction of the FD.^11^ Our results are consistent with this observation showing a significant decrease of FD after a loading dose of three monthly anti-VEGF treatments (Fig. 2). We also demonstrated that the relative difference of FD after the loading dose had a weak discriminative ability regarding the response to anti-VEGF, assessed by retinal fluids evaluation. On a cohort of 36 eyes, Coscas et al. showed a high rate (33.3%) of false-positive qualitative evaluations on OCT-A (i.e., potentially active CNV according to the presence of OCT-A criteria but without retinal fluids according to the OCT B-scan).^8^ Our results agree with this notion of a weak association between blood flow aspect and retinal fluid and suggest that factors other than CNV morphology are responsible for retinal exudation. Regarding the visual acuity at 12 months’ follow-up, FD did not differ between good and bad responders. Moreover, the corresponding GLM models attained a weak discriminative ability to identify patients that had a gain of at least five ETDRS letters of BCVA. The present analysis disclosed that only the BCVA difference after the loading dose and baseline had a good discriminative ability. This agrees with a recent subanalysis of the HARBOR clinical trial spectral domain structural OCT-B scan data performed by Schmidt-Erfurth et al.^24^ Using machine learning, the authors found that functional outcomes were determined by BCVA levels during the loading dose with a minor influence of fluid-related features. Our analysis shows a poor association between the most studied quantitative OCTA parameters and functional outcomes. Concerning the treatment burden, baseline FD had an inverse association with the number of IVI and the respective model had a good discriminative ability to distinguish patients that had seven or less IVI in the first year of a TAE protocol.

Lacunarity is a measure of the size of gaps within a structure and enable to characterize the texture of vascular networks.^18^ Higher values reflect inhomogeneity and lower values reflect a more homogenous vascular structure. Therefore, we would expect naive CNV membranes to show higher lacunarity values after the anti-VEGF treatments. However, our results showed consistently nonsignificant lacunarity differences over the follow-up visits. Roberts et al. have studied patients under treatment for active nAMD with a median of 34 anti-VEGF injections before study inclusion and compared lacunarity between good and poor responders to anti-VEGF.^13^ The authors have also shown nonsignificant differences and hypothesized that the lack of differences was the result of a previous “normalization” of the CNV vasculature by anti-VEGF. In our study, this limitation was overcome by enrolling only treatment-naïve patients. Our findings support the hypothesis that lacunas of the vascular skeleton do not change their arrangement after anti-VEGF, which makes lacunarity a nonrelevant OCT-A parameter for nAMD follow-up.

Blood flow area is a readily available and well-studied OCT-A parameter. Previous studies have shown that OCT-A is able to show the minimal CNV surface^25^ and is reproducible for accurate evaluation of CNV blood flow area.^26^ Miere et al. have recently demonstrated a reduction in CNV surface area in treatment-naïve eyes after the loading dose of three monthly IVI of anti-VEGF.^27^ Our results agree with these findings, as we demonstrated a blood flow area reduction after the loading dose. However, consistent data about the relative modification of flow area during the follow-up of the disease are scarce as most of previous studies had variable follow-up intervals and flexible treatment protocols.^6^^,^^28^ McClintic et al. were the first group to prospectively evaluate a cohort of patients followed over a strict as-needed regimen for 12 months. They verified a significant decrease of CNV blood flow area after the first treatments followed by a regrowth of the lesion. The relative change from baseline was statistically nonsignificant and growth occurred irrespective of retinal fluids in structural OCT.^9^ We verified the same modifications of blood flow area in patients followed under a treat-and-extend regimen. We corroborate the notion that CNVs grow during anti-VEGF treatments. This process had been previously hypothesized by Spaide as “vascular abnormalization.” According to this hypothesis, CNV grow because of unimpeded arteriogenesis in the face of periodic pruning of angiogenic vascular sprouts by VEGF withdrawal.^22^ We have studied whether the relative modifications of surface area could be associated with clinical outcomes. We verified that the ability to distinguish patients with a gain of at least five ETDRS letters in the first year was consistently weak. Although we agree with the notion of vascular “abnormalization,” blood flow area seems to have a minor association with functional outcomes, highlighting the need to assess other parameters. Finally, we verified that baseline blood flow area had an inverse association with the number of IVI and a good discriminative ability to distinguish patients that had seven or less IVI in the first year of a TAE protocol, an association that we have also observed concerning baseline FD. Analyzed together, our results indicate that patients with a lower baseline FD and a lower surface area have higher odds of having eight or more IVI in the first year of a treat-and-extend protocol. While speculative, we hypothesize that this finding reflects the impact of distinct angiogenic environments in CNV morphology. In the presence of high VEGF levels, CNVs would have numerous tiny branches and a disorganized architecture, reflecting an aggressive angiogenic process with greater exudation and a heavier treatment burden. In eyes with lower VEGF availability, CNV would grow without leakage maturing their branching architecture toward a tangled network before exudation becomes overly symptomatic. Figure 4 depicts typical examples of patients that required less than eight IVI in the first year of treatment; we observed large and complex lesions. On the other hand, Figure 5 depicts typical examples of patients that required more than eight IVI in the first year of treatments: we observed small CNVs with a disorganized architecture. According to our hypothesis, there could be a structural-functional relationship between CNV morphology and treatment burden: eyes with lower VEGF production would need less IVI to control the exudative features and the opposite in eyes with higher VEGF production.

Strengths of our study include the use of an automatic image analysis protocol with excellent reproducibility results, enrollment of naïve nAMD patients, and a uniform follow-up evaluation of patients treated according to a treat-and-extend protocol on a real-life setting.

Limitations include a small sample size and technical limitations inherent to the current technology. By conducting a real-world study, 25% of patients were lost to follow-up, giving rise to a selection bias as only the compliant patients remained in the study. This rate of lost on follow-up is similar to other real-life cohorts^29^ and was mostly related to patients’ preference to perform treatments closer to their residence. We acknowledge that including the first eye diagnosed with nAMD per patient might have left out very early presenters. The replication of our findings in very early presenters could be evaluated in future works. We also acknowledge that PED height is associated with signal loss in OCTA.^30^ Selecting patients with PED inferior to 250 µm might have excluded patients in which blood flow was not detectable with OCTA, limiting the application of our results to nAMD patients in which blood flow is readily detected by OCTA. We are also aware of the importance of structural OCT outer retina modifications in response to anti-VEGF. Further studies may evaluate the association between OCT-A parameters, structural OCT changes and functional response. A larger sample size will certainly yield more statistical power to uncover other relationships not identified in the present study.

In conclusion, all the OCTA parameters evaluated were a poor biomarker in predicting anatomic and functional response. Baseline FD and SA were the best biomarkers regarding treatment burden. It appears useful to include baseline FD analysis and blood flow area evaluation in future studies because they seem to be associated with a distinct number of anti-VEGF injections in the first year of disease management.

## Supplementary Material

Supplement 1

Supplement 2
