# Supplementary material for: Quantitative Optical Coherence Tomography Angiography Biomarkers in a Treat-and-Extend Dosing Regimen in Neovascular Age-Related Macular Degeneration
Source: Transl Vis Sci Technol. 2020 Feb 14;9(3):18. doi: 10.1167/tvst.9.3.18 (PMC7351878; doi:10.1167/tvst.9.3.18)
Supplement: Supplement 1 [file tvst-9-3-18_s001.docx]

# Supplementary Figures

**Supplementary Figure S1. Diagram of the observation protocol ad study outcomes.** The treatment protocol consisted in a loading dose of three-monthly IVI of anti-VEGF (aflibercept) after nAMD diagnosis followed by a treat and extend treatment protocol (TAE). All patients had a comprehensive ophthalmic examination at baseline, at three-month visit [3M] (one month after the last of three-monthly intravitreal injections) and at 12-month [12M] follow-up (twelfth month after the first IVI). Outcome 1, treatment response after the loading dose, was evaluated at the 3M visit using structural OCT characteristics. Outcome 2 (functional response) and outcome 3 (treatment burden) were evaluated at the 12M visit concerning the difference in BCVA from baseline and total number of intravitreal injections, respectively.

# Supplementary Tables

**Supplementary Table S1.** Clinical and OCT-A parameters among follow-up evaluations.

| **Parameter** | **Baseline** | **3 M^*^** | **Difference 3M- baseline**^†^ | **12M**^‡^ |
| --- | --- | --- | --- | --- |
| BCVA | 60 (50-73) | 72 (56-80) | 5 (-1-14) | 73 (53-80) |
| CMT | 379 (315-435) | 272 (253-314) | -79 (-161--21) | 286 (263-313) |
| FD | 1.49 (1.40-1.55) | 1.44 (1.25-1.53) | -0.009(-0.07-0.029) | 1.47 (1.37-1.54) |
| LAC | 0.29 (0.26-0.34) | 0.29 (0.26-0.33) | -0.04(-0.08-0.24) | 0.32 (0.27-0.35) |
| SA | 0.62 (0.28-1.52) | 0.47 (0.14-1.43) | -0.07(1-0.07-1.16) | 0.92 (0.35-2,08) |
| VD | 0.55 (0.49-0.67) | 0.50 (0.42-0.60) | -0.05(-0.05-0.10) | 0.53 (0.44-0.61) |

Results are presented as median (interquartile range). *One month after the last of three-monthly injections (n=60) ^†^BCVA and CMT differences were calculated as absolute differences and the other parameters calculated as relative differences ^‡^12 months after the first intravitreal injection (n=45). M: months. Relative difference 3M-baseline = [(value after loading dose – baseline value)/baseline value]. BCVA = best corrected visual acuity. CMT = central macular thickness FD = fractal dimension. LAC = Lacunarity. SA = blood flow area. VD = vessel density.

**Supplementary Table S2. OCT-A parameters at different follow-up times and anatomical response after the loading dose of anti-VEGF treatment.** A good responder was defined as the complete resolution of the intraretinal fluid, subretinal fluid or more than 100μm decrease of central macular thickness one month following the last intravitreal injection of the loading dose. A bad responder was defined as having an increase of central macular thickness or a decrease of less than 100μm in the same time point.

| **Parameter** | **Follow-up time** | **Good anatomical response (n=40)** | **Bad anatomical response (n=24)** | ***p*** |
| --- | --- | --- | --- | --- |
| FD | Baseline | 1.48 (1.39-1.54) | 1.49 (1.43-1.56) | 0.305 |
|  | Relative difference | 0.0007 (-0.05-0.06) | -0.17 (-0.03-0.02) | 0.299 |
| LAC | Baseline | 0.29 (0.25-0.33) | 0.30 (0.27-0.33) | 0.506 |
|  | Relative difference | 0.09 (-0.08-0.28) | -0017 (-0.09-0.12) | 0.172 |
| SA | Baseline | 0.54 (0.20-1.60) | 0.74 (0.36-1.45) | 0.339 |
|  | Relative difference | -0.07 (-0.29-2.04) | -0.09 (-0.38-0.61) | 0.397 |
| VD | Baseline | 0.57 (0.47-0.68) | 0.50 (0.43-0.58) | 0.124 |
|  | Relative difference | -0.05 (-0.21-0.10) | -0.03 (-0.11-0.13) | 0.551 |

Results are presented as median (interquartile range). M = months. FD = Fractal dimension. LAC = lacunarity. SA = choroidal vascularization surface area. VD = vascular density.

**Supplementary Table S3. OCT-A parameters at different follow-up times and functional response at 12-month follow up.** A good functional response was defined as a gain in the best corrected visual acuity of at least five ETDRS letters between baseline and the 12-month visit.

| **Parameter** | **Follow-up time** | **Good functional response (n=33)** | **Bad functional response (n=19)** | ***p*** |
| --- | --- | --- | --- | --- |
| FD | Baseline | 1.49 (1.39-1.56) | 1.51 (1.42-1.53) | 0.887 |
|  | Relative difference | -0.01 (-0.05-0.02) | -0.001 (-0.07-0.05) | 0.902 |
| LAC | Baseline | 0.30 (0.25-0.35) | 0.29 (0.26-0.34) | 0.887 |
|  | Relative difference | 0.04 (-0.05-0.021) | 0.01 (-0.17-0.41) | 0.697 |
| SA | Baseline | 0.64 (0.24-2.01) | 0.55 (0.32-1.45) | 0.697 |
|  | Relative difference | -0.05 (-0.30-1.22) | -0.15 (-0.39-0.98) | 0.537 |
| VD | Baseline | 0.57 (0.44-0.69) | 0.57 (0.45-0.64) | 0.283 |
|  | Relative difference | -0.05 (-0.27-0.07) | -0.04 (-0.09-0.13) | 0.377 |

Results are presented as median (interquartile range). M = months. FD = Fractal dimension. LAC = lacunarity. SA = choroidal vascularization surface area. VD = vascular density.

**Supplementary Table S4. OCT-A parameters at different follow-up times and treatment burden.** Regular treatment was considered for patients with eight or more IVI and extended treatment was considered for patients with seven or less injections in the first twelve months.

| **Parameter** | **Follow-up time** | **Regular Treatment (n=23)** | **Extended treatment (=29)** | ***p*** |
| --- | --- | --- | --- | --- |
| FD | Baseline | 1.40 (1.31-1.49) | 1.52 (1.47-1.56) | 0.001 |
|  | Relative difference | 0.01 (-0.06-0.08) | -0.03 (-0.07-0.01) | 0.023 |
| LAC | Baseline | 0.28 (0.23-0.35) | 0.30 (0.27-0.34) | 0.111 |
|  | Relative difference | 0.02 (-0.07-0.41) | 0.04 (-0.08-0.21) | 0.537 |
| SA | Baseline | 0.33 (0.17-0.58) | 1.14 (0.48-2.80) | 0.001 |
|  | Relative difference | 0.26 (-0.23-3.56) | -0.15 (-0.43-0.60) | 0.050 |
| VD | Baseline | 0.64 (0.17-0.58) | 0.48 (0.43-0.60) | 0.009 |
|  | Relative difference | -0.05 (-0.32-0.11) | -0.01 (-0.10-0.11) | 0.315 |

Result are presented as median (interquartile range). M = months. FD = Fractal dimension. LAC = lacunarity. SA = choroidal vascularization surface area. VD = vascular density.

**Supplementary Table S5.** Models’ performance analysis regarding the anatomic response to anti-VEGF. A good responder was defined as complete resolution of the intraretinal macular edema, subretinal fluid and pigment epithelial detachment or more than 100 μm decrease of CMT one month following the last intra vitreal injection of the loading dose.

| **Parameter** | **AUC** | **95% CI** |
| --- | --- | --- |
| FD (Baseline) | 0.577 | 0.40-0.73 |
| LAC (Baseline) | 0.550 | 0.47-0.78 |
| SA (Baseline) | 0.572 | 0.40-0.72 |
| VD (Baseline) | 0.616 | 0.45-0.77 |
| FD (relative difference) | 0.567 | 0.43-0.75 |
| LAC (relative difference) | 0.604 | 0.46-0.77 |
| SA (relative difference) | 0.521 | 0.41-0.73 |
| VD (relative difference) | 0.595 | 0.39-0.72 |
| FD (after loading dose) | 0.564 | 0.42-0.73 |
| LAC (after loading dose) | 0.652 | 0.49-0.79 |
| SA (after loading dose) | 0.539 | 0.36-0.67 |
| VD (after loading dose) | 0.520 | 0.35-0.66 |

FD = Fractal dimension. LAC = Lacunarity. SA = blood flow surface area. VD = Vessel Density. AUC = Area Under the Receiver Operating Characteristic Curve. CI = Confidence Interval. AUCs and corresponding 95% CI were estimated using generalized linear models.

**Supplementary Table S6.** Models’ performance analysis regarding functional response after twelve months. A good functional response was defined as a gain in the BCVA of at least five ETDRS letters (superior or equal to vs inferior to).

| **Parameter** | **AUC** | **95%CI** |
| --- | --- | --- |
| FD (Baseline) | 0.512 | 0.35-0.67 |
| LAC (Baseline) | 0.512 | 0.35-0.68 |
| SA (Baseline) | 0.467 | 0.37-0.69 |
| VD (Baseline) | 0.590 | 0.44-0.75 |
| FD (relative difference) | 0.616 | 0.41-0.73 |
| LAC (relative difference) | 0.592 | 0.35-0.71 |
| SA (relative difference) | 0.574 | 0.38-0.72 |
| VD (relative difference) | 0.509 | 0.34-0.68 |
| FD (after loading dose) | 0.590 | 0.42-0.76 |
| LAC (after loading dose) | 0.593 | 0.44-0.76 |
| SA (after loading dose) | 0.568 | 0.38-0.72 |
| VD (after loading dose) | 0.603 | 0.45-0.76 |

FD = Fractal dimension. LAC = Lacunarity. SA = blood flow surface area. VD = Vessel Density. AUC = Area Under the Receiver Operating Characteristic Curve. CI = Confidence Interval. AUCs and corresponding 95% CI were estimated using generalized additive models.

**Supplementary Table S7.** Models’ performance analysis regarding treatment burden in the first twelve months. Seven intravitreal injections were considered as a cut-off (inferior or equal to vs superior to).

| **Parameter** | **AUC** | **95%CI** |
| --- | --- | --- |
| FD (Baseline) | 0.7766 | 0.64-0.91 |
| LAC (Baseline) | 0.6297 | 0.47-0.78 |
| SA (Baseline) | 0.7616 | 0.63-0.90 |
| VD (baseline) | 0.7121 | 0.57-0.86 |
| FD (relative difference) | 0.6342 | 0.44-0.76 |
| LAC (relative difference) | 0.6702 | 0.39-0.71 |
| SA (relative difference) | 0.6852 | 0.51-0.81 |
| VD (relative difference) | 0.5217 | 0.42-0.75 |
| FD (after loading dose) | 0.5052 | 0.35-0.66 |
| LAC (after loading dose) | 0.5712 | 0.41-0.73 |
| SA (after loading dose) | 0.5157 | 0.32-0.65 |
| VD (after loading dose) | 0.6012 | 0.41-0.73 |

FD = Fractal dimension. LAC = Lacunarity. SA = blood flow surface area. VD = Vessel Density. AUC = area under the Receiver Operating Characteristic Curve. CI = Confidence Interval. AUCs and corresponding 95% CI were estimated using generalized additive models.

**Supplementary Table S8.** The intraclass correlation coefficient between the two consecutive baseline acquisitions for the four quantitative parameters.

| **Parameter** | **Acquisition 1**  **Mean (SD)** | **Acquisition 2**  **Mean (SD)** | **ICC (95% CI)** |
| --- | --- | --- | --- |
| FD | 1.472 (0.098) | 1.469 (0.098) | 0.995 (0.992-0.997) |
| LAC | 0.292 (0.057) | 0.289 (0.0527) | 0.965 (0.942-0.979) |
| SA | 1.300 (1.788) | 1-283 (1.717) | 0.991 (0.985-0.994) |
| VD | 0.556 (0.169) | 0.571 (0.179) | 0.929 (0.885-0.956) |

SD = standard deviation. ICC = intraclass correlation coefficient. CI = confidence interval. FD = fractal dimension. LAC = Lacunarity. SA = blood flow surface area. VD = vessel density.
