# Supplementary figures and images for: Quantitative Optical Coherence Tomography Angiography Biomarkers in a Treat-and-Extend Dosing Regimen in Neovascular Age-Related Macular Degeneration
Source: Transl Vis Sci Technol. 2020 Feb 14;9(3):18. doi: 10.1167/tvst.9.3.18 (PMC7351878; doi:10.1167/tvst.9.3.18)

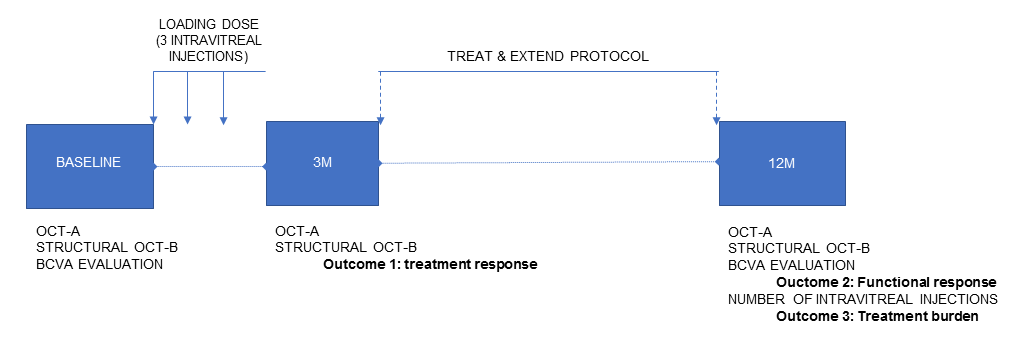

Supplement: Supplement 2 [file tvst-9-3-18_s002.png]
